# Supplementary material for: Clinical Interventions and Inflammatory Signaling Shape the Transcriptional and Cellular Architecture of the Early Postnatal Lung
Source: bioRxiv. 2025 Oct 17:2025.10.17.683116. Preprint. [Version 1] doi: 10.1101/2025.10.17.683116 (PMC12633012; doi:10.1101/2025.10.17.683116)
Supplement: 1 — Figure S1. Subclustering of Major Cell Classes and Identification of Known Cell Types in the Early Postnatal Lung (A) UMAP embedding of mesenchymal subclusters, annotated based on enrichment of known marker genes shown in (B). (B) Dot plot illustrating shared and distinguishing markers of alveolar fibroblast-c1, -c2, and -c3, subclusters, adventitial fibroblasts, alveolar myofibroblasts, pericytes, and shared marker expression across intermediate fibroblasts. (C) UMAP embedding of endothelial subclusters with annotations based on known marker enrichment highlighted in (D). (D) Dot plot showing markers distinguishing capillary aerocytes and capillary endothelial cells, and distinguishing markers among larger-vessel endothelial populations (venous, arterial, lymphatics). (E) UMAP embedding of immune subclusters with annotations based on marker enrichment shown in (F). (F) Dot plot illustrating shared and distinguishing markers across lymphoid and myeloid populations. (G) UMAP embedding of epithelial subclusters annotated by marker expression shown in (H). (H) Dot plot showing markers distinguishing epithelial populations including AT1, AT2, AT2 to AT1 transitional, basal, multiciliated, respiratory bronchiole, and pulmonary neuroendocrine (PNEC) cells. (I) Dot plot showing genes enriched in TREM2+ macrophages indicative of an activated or polarized state. (J) UMAP of PNEC subclusters enriched for GRP or GHRL, with feature and scatter plots demonstrating mutually exclusive expression of subset markers in the early postnatal distal lung. (K) Dot plot showing that multiciliated cells exhibit a C6+/MUC16+ transcriptional profile in the early postnatal distal lung. (L) Hematoxylin-and-eosin staining of fetal (14–16 weeks), early postnatal (birth to two years), and adult alveoli. Scale bar = 50 μm. (M) Comparison of cellular composition between 14–16-week fetal and early postnatal distal lung by major cell class, highlighting the developmental shift from mesenchymal to epit [file NIHPP2025.10.17.683116V1-supplement-1.pdf]

Frum et al., 2025 Figure S1

available under aCC-BY 4.0 International license.

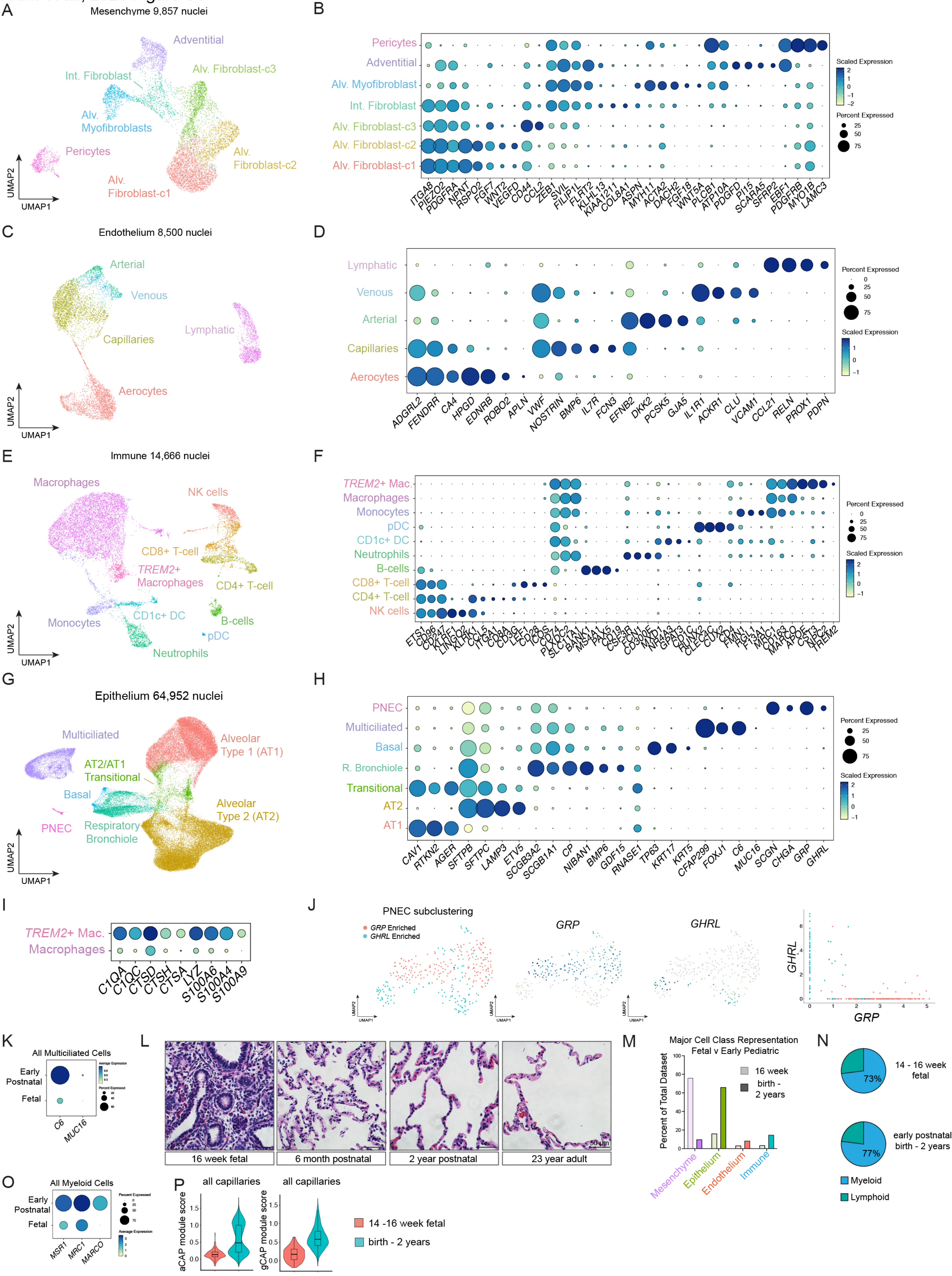

bioRxiv preprint doi: https://doi.org/10.1101/2025.10.17.683116; this version posted October 17, 2025. The copyright holder for this preprint (which was not certified by peer review) is the author/funder, who has granted bioRxiv a license to display the preprint in perpetuity. It is made available under aCC-BY 4.0 International license.

Figure S1. Subclustering of Major Cell Classes and Identification of Known Cell Types in the Early Postnatal Lung
(A) UMAP embedding of mesenchymal subclusters, annotated based on enrichment of known marker genes shown in (B).
(B) Dot plot illustrating shared and distinguishing markers of alveolar fibroblast-c1, -c2, and -c3, subclusters, adventitial fibroblasts, alveolar myofibroblasts, pericytes, and shared marker expression across intermediate fibroblasts.
(C) UMAP embedding of endothelial subclusters with annotations based on known marker enrichment highlighted in (D).
(D) Dot plot showing markers distinguishing capillary aerocytes and capillary endothelial cells, and distinguishing markers among larger-vessel endothelial populations (venous, arterial, lymphatics).
(E) UMAP embedding of immune subclusters with annotations based on marker enrichment shown in (F).
(F) Dot plot illustrating shared and distinguishing markers across lymphoid and myeloid populations.
(G) UMAP embedding of epithelial subclusters annotated by marker expression shown in (H).
(H) Dot plot showing markers distinguishing epithelial populations including AT1, AT2, AT2 to AT1 transitional, basal, multiciliated, respiratory bronchiole, and pulmonary neuroendocrine (PNEC) cells.
(I) Dot plot showing genes enriched in *TREM2*+ macrophages indicative of an activated or polarized state.
(J) UMAP of PNEC subclusters enriched for *GRP* or *GHRL*, with feature and scatter plots demonstrating mutually exclusive expression of subset markers in the early postnatal distal lung.
(K) Dot plot showing that multiciliated cells exhibit a *C6*+/*MUC16*+ transcriptional profile in the early postnatal distal lung.
(L) Hematoxylin-and-eosin staining of fetal (14–16 weeks), early postnatal (birth to two years), and adult alveoli. Scale bar = 50 μm.
(M) Comparison of cellular composition between 14–16-week fetal and early postnatal distal lung by major cell class, highlighting the developmental shift from mesenchymal to epithelial dominance.
(N) Relative myeloid and lymphoid contributions to the immune compartment are similar between fetal and early postnatal specimens.
(O) Dot plot comparing macrophage marker expression between fetal and early postnatal lungs reveals emergence of *MARCO*+ resident macrophages postnatally.
(P) Violin plot comparing aCAP (aerocyte) and gCAP (capillary) module scores, derived from Travaglini et al. 2020, across all capillary cells in fetal and early postnatal datasets, demonstrating the emergence of specialized capillary signatures, particularly aCAP/aerocyte enrichment, after birth.

Frum et al., 2025 Figure S2

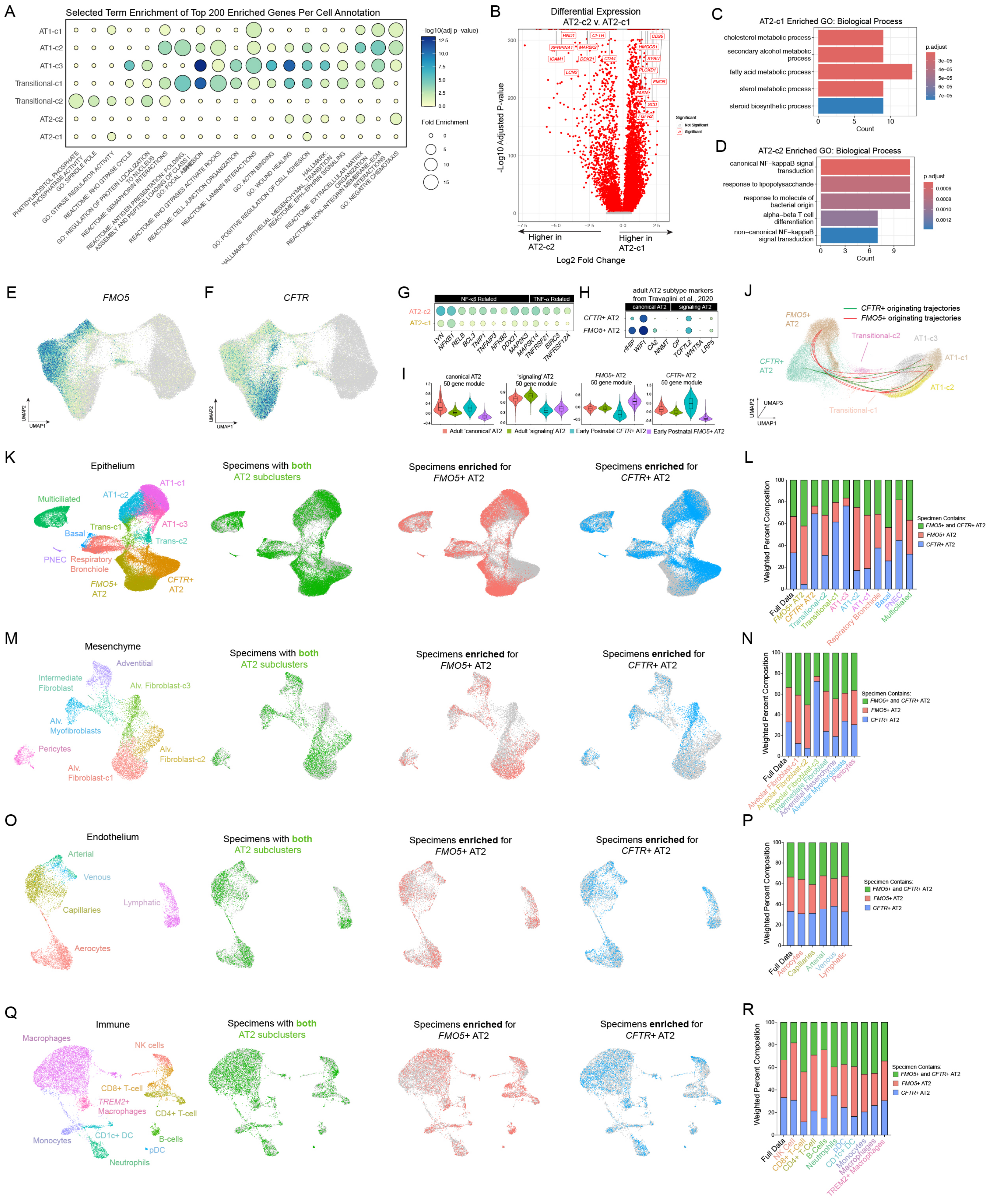

bioRxiv preprint doi: https://doi.org/10.1101/2025.10.17.683116; this version posted October 17, 2025. The copyright holder for this preprint (which was not certified by peer review) is the author/funder, who has granted bioRxiv a license to display the preprint in perpetuity. It is made available under aCC-BY 4.0 International license.

Figure S2. Functional Divergence and Cell Type Associations of AT2 Subclusters in the Early Postnatal Lung

(A) Dot plot of gene set enrichment analysis showing biological processes and pathways enriched across alveolar epithelial subclusters, emphasizing terms enriched in AT2 to AT1 transitional and AT1 clusters.
(B) Volcano plot showing significantly differentially expressed genes between AT2 subclusters.
(C) The top five enriched GO Biological Process terms for AT2-c1 markers converge on lipid metabolic processes.
(D) The top five enriched GO Biological Process terms for AT2-c2 markers converge on immune-responsive pathways, including NF-κB and other immune related signaling.
(E) Feature plot showing the spatial distribution of the AT2-c1 marker *FMO5*.
(F) Feature plot showing the spatial distribution of the AT2-c2 marker *CFTR*.
(G) Dot plot showing enrichment of known targets of NF-κB and TNF-α signaling in AT2-c2, supporting an immune-responsive phenotype in AT2-c2 cells.
(H) Dot plot showing expression of adult AT2 subtype markers[27] within early postnatal *FMO5*+ and *CFTR*+ AT2 populations.
(I) Gene-module scoring comparing early postnatal and adult datasets. Left: scoring of early postnatal AT2 subclusters using 50-gene modules defining canonical and signaling AT2 subtypes in the adult lung. Right: scoring of adult AT2 subclusters using 50-gene modules derived from early postnatal *FMO5*+ and *CFTR*+ AT2 transcriptional axes.
(J) Slingshot trajectory analysis on a three-dimensional UMAP of alveolar epithelial cells reveals differentiation trajectories originating from *FMO5*+ and *CFTR*+ AT2 subtypes and converging on AT1 cells through AT2 to AT1 transitional intermediates.
(K) Epithelial UMAP showing annotations and overlaying cells from specimens classified as having comparable proportions of both AT2 subtypes (green), enriched for AT2-c1 (red), or enriched for AT2-c2 (blue).
(L) Weighted composition of epithelial cell types across specimen classifications, adjusted so that each category contributes equally to the overall epithelial compartment (left column, “Full Data”).
(M) Mesenchymal UMAP showing annotations and overlaying cells from the same specimen categories (green = both AT2 subtypes, red = AT2-c1 enriched, blue = AT2-c2 enriched).
(N) Weighted composition of mesenchymal cell types across specimen classifications, normalized as in (L).
(O) Endothelial UMAP showing annotations and specimen overlays as in (M).
(P) Weighted composition of endothelial cell types across specimen classifications, normalized as in (L).
(Q) UMAP of immune cell subclustering showing annotations and specimen overlays as in (M).
(R) Weighted composition of immune cell types across specimen classifications, normalized as in (L).

Frum et al., 2025 Figure S3

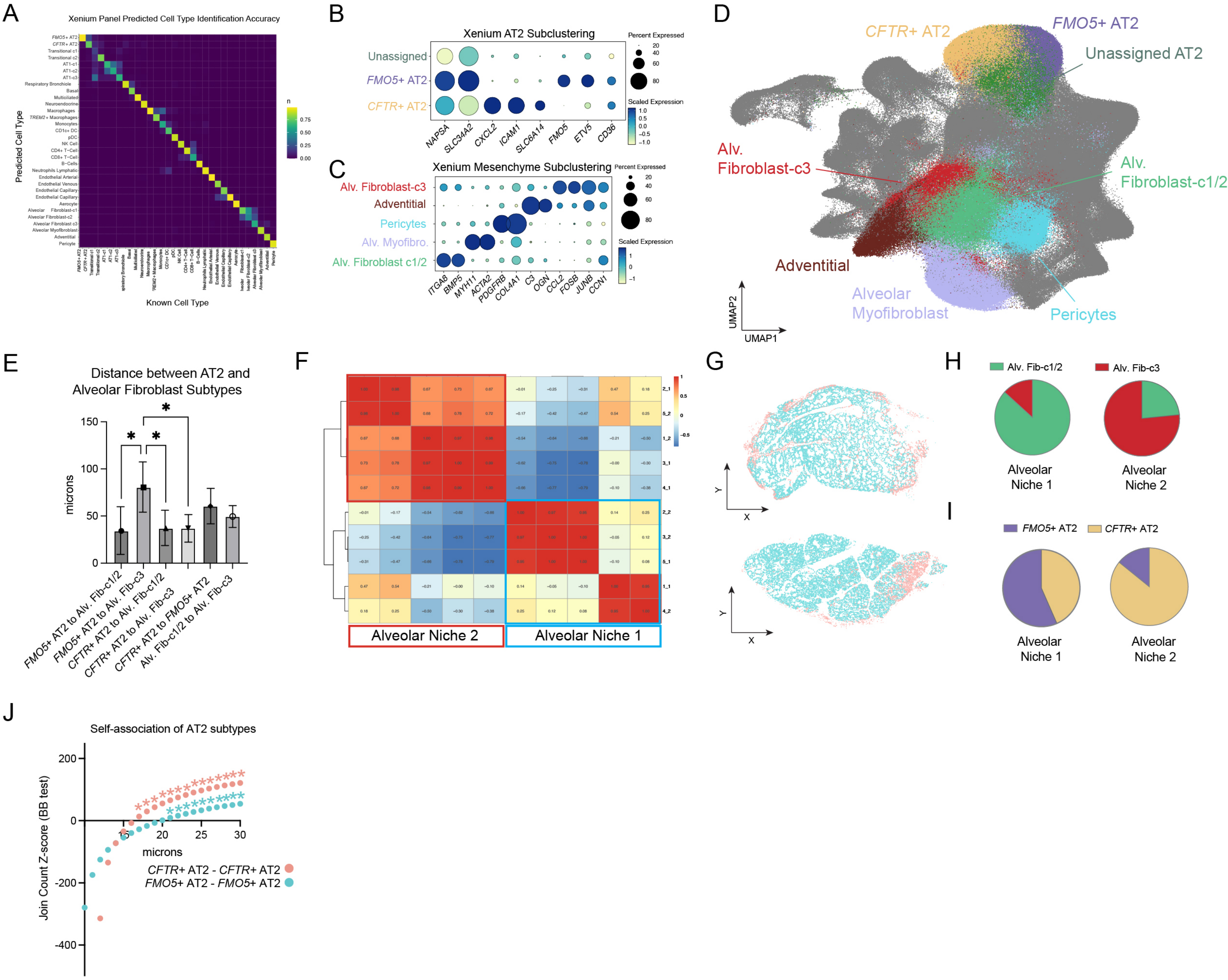

bioRxiv preprint doi: https://doi.org/10.1101/2025.10.17.683116; this version posted October 17, 2025. The copyright holder for this preprint (which was not certified by peer review) is the author/funder, who has granted bioRxiv a license to display the preprint in perpetuity. It is made available under aCC-BY 4.0 International license.

Figure S3. *FMO5*+ and *CFTR*+ AT2s are Spatially Associated with Distinct Alveolar Fibroblast Subtypes
(A) Confusion matrix showing the accuracy of cell type identification using the custom 480-gene Xenium probe set (y-axis), validated against cell type identities from early postnatal snRNA-seq data (x-axis).
(B) Dot plot showing marker gene expression for manually annotated AT2 cells in the Xenium dataset. This approach identifies a subset of unassigned AT2 cells lacking FMO5 and CFTR expression, which were excluded from analyses in panels EJ.
(C) Dot plot showing marker gene expression for manually annotated mesenchymal cells in the Xenium dataset, distinguishing alveolar fibroblast-c3 from alveolar fibroblast-c1/2 populations.
(D) Integrated UMAP of all segmented spatial transcriptomic cells, colored by manually annotated cell type identities.
(E) Quantification of the average spatial distance between AT2 and alveolar fibroblast subtypes across tissue sections. FMO5+ AT2 cells are positioned farther from alveolar fibroblast-c3 cells than from alveolar fibroblast-c1/2 cells, whereas CFTR+ AT2 cells are closely associated with alveolar fibroblast-c3 cells.
(F) Heatmap showing Pearson's correlation of niche cell composition derived from Seurat v5's k-means–based niche analysis using only manually annotated AT2 and mesenchymal cells. Two distinct alveolar niche clusters emerge, corresponding to the FMO5+ and CFTR+ AT2 microenvironments.
(G) Spatial maps of two early postnatal lung sections colored by niche classification based on (F).
(H) Pie charts showing that alveolar niche 1 is enriched for and alveolar fibroblast-c1/2 cells, while alveolar niche 2 is enriched for alveolar fibroblast-c3 cells.
(I) Pie charts showing that alveolar niche 1 is enriched for *FMO5*+ AT2 cells and *CFTR*+ AT2 cells.
(J) Join count spatial autocorrelation analysis of AT2 subtype self-association. Z-scores were calculated across increasing spatial distances. Asterisks indicate significant positive association (*$p < 0.05$). CFTR+ AT2 cells show stronger local self-association than FMO5+ AT2 cells, indicating tighter spatial clustering.

Frum et al., 2025 Figure S4

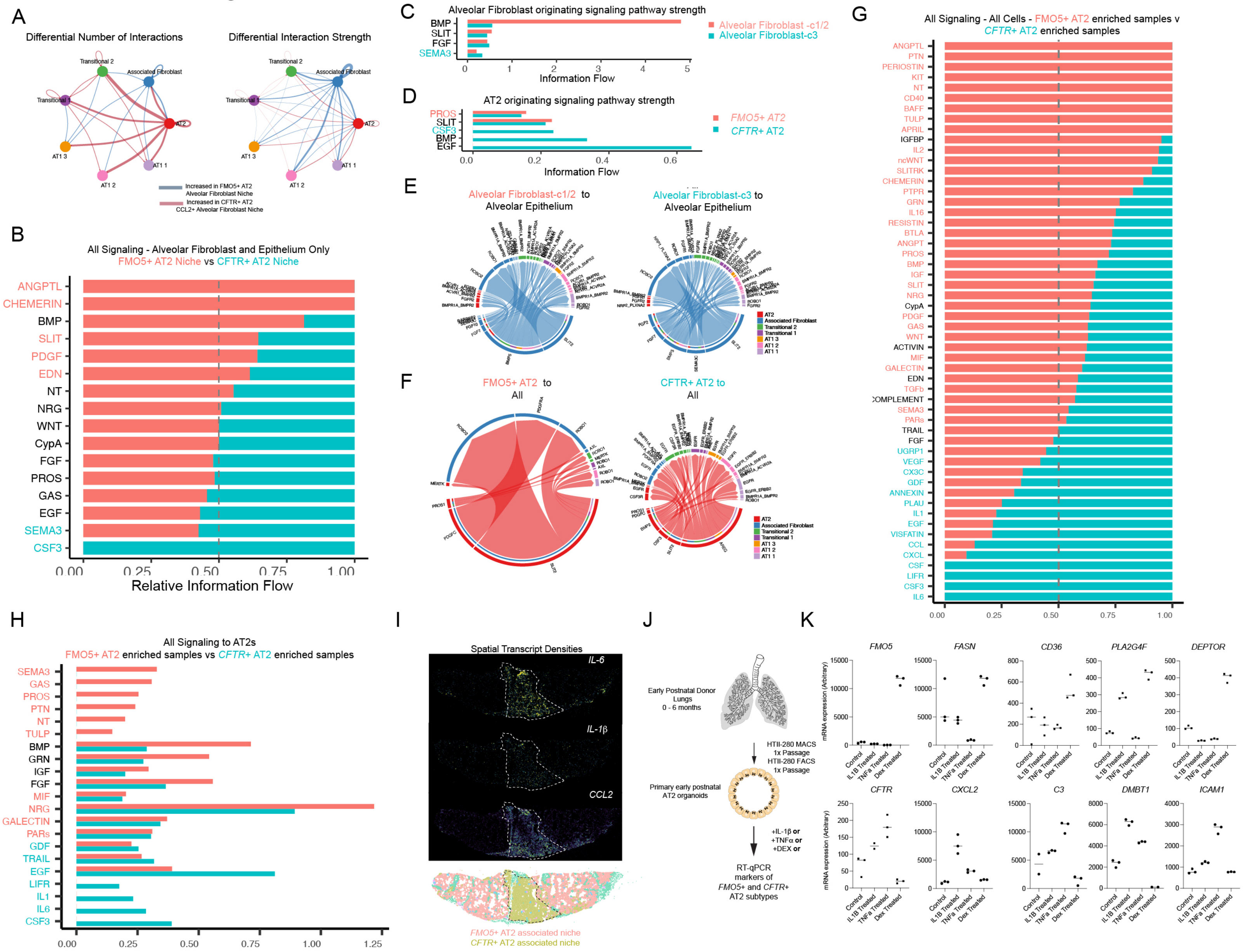

bioRxiv preprint doi: https://doi.org/10.1101/2025.10.17.683116; this version posted October 17, 2025. The copyright holder for this preprint (which was not certified by peer review) is the author/funder, who has granted bioRxiv a license to display the preprint in perpetuity. It is made available under aCC-BY 4.0 International license.

Figure S4. CellChat Analysis of *FMO5*+ and *CFTR*+ AT2 alveolar niches and recapitulation of *FMO5*+ and *CFTR*+ AT2 States in Primary Early Postnatal Organoids

(A–F) CellChat analysis of alveolar epithelial and mesenchymal interactions within *FMO5*+ and *CFTR*+ AT2 niches. The *FMO5*+ AT2 niche includes *FMO5*+ AT2 cells, alveolar fibroblast-c1/2 populations, and all AT2 to AT1 transitional and AT1 subclusters. The *CFTR*+ AT2 niche includes *CFTR*+ AT2 cells, alveolar fibroblast-c3 populations, and the same transitional and AT1 subclusters as the *FMO5*+ AT2 niche. AT2 subclusters and their associated fibroblast subsets (i.e. *CFTR*+ AT2 and AF-c3) are referred to as “AT2” and “Associated Fibroblast” in the plots.

(A) Differential number of interactions and interaction strength between alveolar epithelial and mesenchymal cell types in each niche.

(B) Comparison of pathway-level signaling activity between *FMO5*+ and *CFTR*+ AT2 niches. Pathways highlighted in red are significantly more active in the *FMO5*+ AT2 niche, whereas those in blue are more active in the *CFTR*+ AT2 niche.

(C) Comparison of absolute outgoing signaling from alveolar fibroblast-c1/2 cells in the *FMO5*+ AT2 niche and alveolar fibroblast-c3 cells in the *CFTR*+ AT2 niche. Pathways highlighted in blue are significantly more active when alveolar fibroblast-c3 present.

(D) Comparison of absolute outgoing signaling from *FMO5*+ and *CFTR*+ AT2 cells within their respective niches. Pathways highlighted in red are more active in the *FMO5*+ AT2 niche; those in blue are more active in the *CFTR*+ AT2 niche.

(E) Circle plot showing all ligand–receptor interactions originating from alveolar fibroblast-c1/2 (left) or alveolar fibroblast-c3 cells (right). Circle size indicates total signaling strength from the source fibroblast; segment size indicates the relative contribution of each ligand or receptor. alveolar fibroblast-c3 cells express more *FGF2* and *SEMA3C* relative to alveolar fibroblast-c1/2 cells.

(F) Circle plot showing all ligand–receptor interactions originating from *FMO5*+ AT2 (left) or CFTR+ AT2 (right) subclusters. *CFTR*+ AT2 cells uniquely express *CSF3* and *AREG*.

(G–H) CellChat comparison between specimens enriched for *FMO5*+ or *CFTR*+ AT2 cells, considering all cell types.

(G) Relative pathway-level signaling activity across all cell types. Pathways in red are more active in the *FMO5*+ AT2 niche, and those in blue are more active in the *CFTR*+ AT2 niche.

(H) Comparison of total incoming signaling strength to AT2 cells between *FMO5*+ AT2- and C*FTR*+ AT2-enriched specimens.

(I) Spatial transcript density maps of *IL-6*, *IL-1*β, and *CCL2* transcripts from Xenium spatial transcriptomics of early postnatal lung sections. Transcripts encoding these ligands are enriched in regions corresponding to the *CFTR*+ AT2-enriched alveolar niche (gold, black dashed outlines).

(J) Experimental scheme for generating early postnatal AT2 organoids from donor lungs and directing them toward *FMO5*+ or *CFTR*+ transcriptional states. Briefly, donor lungs (0–6 months) were dissociated to single cells, AT2s isolated via HTII-280 magnetic-assisted cell sorting, and cultured in AT2 organoid media[85,86] for 30 days. Following one passage and purification by flow-assisted cell sorting for HTII-280+/NGFR- cells, organoids were expended and treated for 10 days with dexamethasone, IL-1β, or TNF-α, then collected for RT-qPCR.

(K) RT-qPCR analysis of *FMO5*+ (top panels) and *CFTR*+ (bottom panels) AT2 marker genes. Primary early postnatal AT2 organoids recapitulate *in vivo* AT2 cell states, with IL-1β, or TNF-α promoting *CFTR*+ AT2 marker expression and dexamethasone inducing *FMO5*+ AT2 marker expression.

Frum et al., 2025 Figure S5

bioRxiv preprint doi: https://doi.org/10.1101/2025.10.17.683116; this version posted October 17, 2025. The copyright holder for this preprint (which was not certified by peer review) is the author/funder, who has granted bioRxiv a license to display the preprint in perpetuity. It is made available under aCC-BY 4.0 International license.

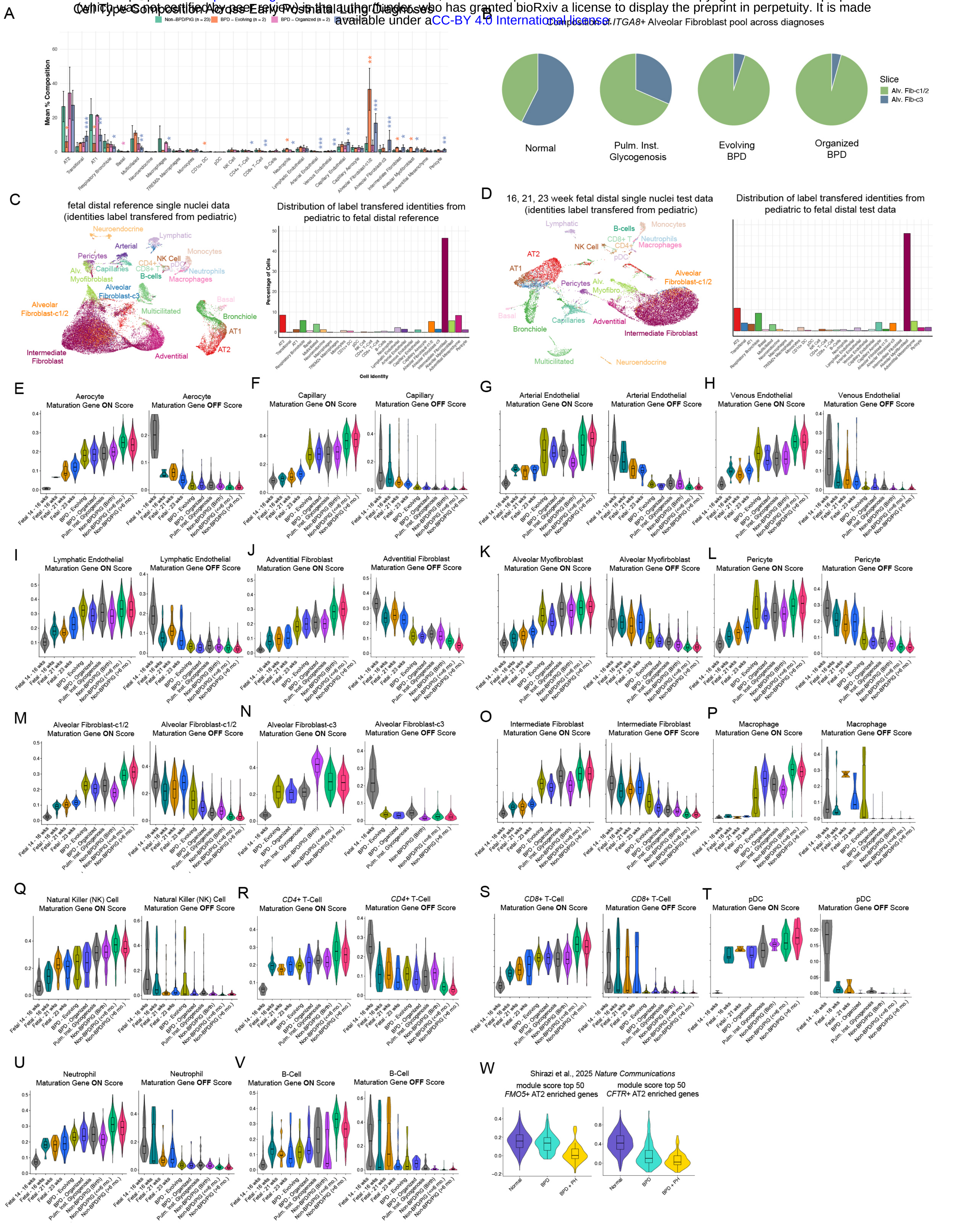

bioRxiv preprint doi: https://doi.org/10.1101/2025.10.17.683116; this version posted October 17, 2025. The copyright holder for this preprint (which was not certified by peer review) is the author/funder, who has granted bioRxiv a license to display the preprint in perpetuity. It is made available under aCC-BY 4.0 International license.

Figure S5. Cell Type Composition and Maturation Scoring Differences Between Fetal, Non-Diagnosed, and Diseased Early Postnatal Lung Specimens

(A) Bar chart comparing mean cell type composition across all non-diagnosed and diagnosed early postnatal lung specimens. Error bars indicate standard deviation. *$p < 0.05$, **$p < 0.01$, ***$p < 0.001$ as determined by Wilcoxon t-test treating each specimen as a biological replicate.

(B) Pie charts showing the proportion of mesenchymal cells mapping to alveolar fibroblast-c1/c2 and alveolar fibroblast-c3 populations in non-diagnosed, pulmonary interstitial glycogenosis (P.I.G.), evolving bronchopulmonary dysplasia (BPD), and organized BPD specimens.

(C) UMAP of 14 to 16-week fetal distal lung specimens used as a reference to define maturation gene sets, with cell type identities transferred computationally from the early postnatal snRNA-seq atlas (left). Bar chart showing the inferred cell type composition of 14 to 16-week fetal distal samples (right).

(D) UMAP of additional 16-, 21-, and 23-week fetal distal lung specimens used as benchmarks for maturation scoring. Identities were transferred computationally from the early postnatal lung atlas (left), and the corresponding inferred cell type composition is shown (right).

(E–V) Violin plots showing module scores for maturation gene programs across major cell types. The “maturation-on” score represents the top 100 genes enriched in early postnatal relative to fetal cells, and the “maturation-off” score represents the top 100 genes enriched in fetal relative to postnatal cells. Panels show scores for:

(E) aerocytes, (F) capillary endothelial, (G) arterial endothelial, (H) venous endothelial, (I) lymphatic endothelial, (J) adventitial fibroblasts, (K) Alveolar Myofibroblasts, (L) pericytes, (M) alveolar fibroblast-c1/c2 cells, (N) alveolar fibroblast-c3 cells, (O) intermediate fibroblasts, (P) macrophages, (Q) natural killer, (R) CD4+ T Cells, (S) CD8+ T Cells, (T) plasmacytoid dendritic cells (pDCs), U) Neutrophils, and V) B Cells.

(W) Violin plots showing module scores for *FMO5*+ and *CFTR*+ AT2 transcriptional signatures in an independent dataset containing BPD and BPD with pulmonary hypertension (BPD + PH) specimens[34]. This data shows the *CFTR*+ AT2 state is reduced in BPD.
